# Experimental Study of the Dynamic Compressive and Tensile Anisotropic Mechanical Properties and Failure Modes of Shale

**DOI:** 10.3390/s25092905

**Published:** 2025-05-04

**Authors:** Qian Dong, Hao Tong, Jinshan Sun, Songlin Peng, Jijie Jia

**Affiliations:** 1 State Key Laboratory of Precision Blasting, Jianghan University, Wuhan 430056, China; sun99001@126.com (J.S.); jiajijie@stu.jhun.edu.cn (J.J.); 2 Hubei Key Laboratory of Blasting Engineering, Jianghan University, Wuhan 430056, China; 3 Hubei (Wuhan) Institute of Explosion and Blasting Technology, Jianghan University, Wuhan 430056, China; 4 China Communications Construction Company Second Harbor Engineering Company Ltd., Wuhan 430040, China; pengsonglin1998@163.com

**Keywords:** shale, dynamic mechanical property, failure mode, anisotropy

## Abstract

To investigate the dynamic compressive and tensile mechanical properties and failure modes of shale, split Hopkinson pressure bar (SHPB) and high-speed imaging and digital image correlation (DIC) technologies were adopted. Dynamic impact compression and Brazilian splitting tests of shale samples at five different bedding angles of 0°, 30°, 45°, 60°, and 90° (angles between the dynamic compressive loading direction or the actual dynamic tensile loading direction and the normal direction of the bedding planes) were conducted to reveal the influence of the bedding angle, strain rate, and impact velocity on the dynamic compressive and tensile mechanical properties and failure modes of shale. The experimental results indicate that the dynamic compressive and tensile strengths, as well as the failure modes, of shale exhibit significant anisotropy. The dynamic strength of the shale increased with the strain rate and impact velocity, while it decreased initially and then increased with the increase in the bedding angle. The failure modes of shale under dynamic compressive and tensile loads are closely related to the bedding angle, strain rate, and impact velocity.

## 1. Introduction

Shale is a sedimentary rock distributed worldwide and has attracted much attention for its unique physical and mechanical properties. As a common layered rock structure, shale exhibits significant anisotropy in its mechanical properties [1]. These mechanical properties make engineering construction in shale formation challenging. Shale is composed of a rock matrix and bedding planes, and the bedding planes are usually weaker and more likely to slip and crack than the rock matrix, thus significantly affecting its strength and stability. Blasting engineering in shale formations involves the specialized discipline of designing and executing controlled explosive techniques to fracture, fragment, or displace shale for applications such as mining, tunneling, oil/gas extraction, or civil construction, and the anisotropic mechanical properties of shale significantly affect the blasting effect and can lead to accidents. Moreover, the bedding planes may also induce the asymmetric expansion of blasting cracks and increase the damage range of shale [2,3]. Hence, it is of great theoretical and practical significance to study the dynamic mechanical properties of shale under impact loads. From a theoretical point of view, the mechanical response mechanism of shale under an impact load is complex, involving stress wave propagation, dynamic crack propagation, energy dissipation, etc. In-depth study of these mechanisms promises to improve rock dynamics theory and provide a theoretical guide for the safe and efficient construction of shale formations. From a practical point of view, the mechanical properties of shale under an impact load directly affect the safety and efficiency of blasting engineering, oil and gas exploitation, underground engineering, and other fields [4,5,6]. The systematic study of the dynamic mechanical properties of shale is important for ensuring engineering safety, improving engineering efficiency and promoting the exploitation and utilization of shale resources.

Various studies have been carried out on the static mechanical properties of shale, which exhibits significant anisotropy owing to its bedding structure, and its parameters, such as compressive/tensile strength, elastic modulus, and Poisson’s ratio, vary with the loading direction [7,8]. These parameters not only illustrate the deformation and failure mechanisms of shale under static loading but also highlight the directional dependence of the mechanical properties on the bedding’s structure [9,10,11,12]. Extensive research has been conducted on the anisotropy of shale’s static mechanical properties. For instance, Niandou et al. [13] investigated the anisotropic mechanical behavior of Tournemire shale through uniaxial and triaxial tests, revealing that both plastic deformation and failure modes are anisotropic. Geng et al. [14] demonstrated that the brittleness anisotropy of Longmaxi shale samples is dependent on the bedding angle, with anisotropy decreasing as the confining pressure increases, based on triaxial experiments under various confining pressures. Heng et al. [15] conducted Brazilian splitting, direct shear, and three-point bending (TPB) tests, finding that the tensile strength, cohesion, internal friction angle, and fracture toughness of the bedding planes are significantly lower than those of the rock matrix. This indicates that bedding planes are quantitatively weaker in tensile strength, shear strength, and fracture toughness, underscoring the pronounced anisotropy of shale. Jin et al. [16] performed uniaxial compression, direct tensile, and Brazilian splitting tests on Marcellus shale samples with different bedding plane orientations, providing in-depth insight into the deformation, strength, and fracture properties of shale, and confirming that the fracture properties of Marcellus shale are also anisotropic. Tavallali et al. [17] conducted a detailed study on the static Brazilian splitting test of laminated sandstone, examining the effects of bedding planes’ direction, shape, and microscopic parameters. They observed that the Brazilian splitting failure mode of laminated sandstone is significantly influenced by the bedding planes, with the tensile strength increasing with the bedding plane angle, demonstrating clear anisotropy. Tien et al. [18] studied the failure criteria of stratified rock masses and found that, regardless of whether the rock mass slips, its strength and deformation modulus are influenced by the bedding angle, varying in a U-shaped or wavy pattern. Wang et al. [19] investigated the damage evolution and acoustic emission (AE) characteristics during the failure process of anisotropic shale, revealing the effects of the angle between the loading direction and the bedding planes.

However, for the blasting and mechanical excavation involved in engineering construction in a widely distributed shale formation, the dynamic mechanical properties of the shale must be clarified. At present, the split Hopkinson pressure bar (SHPB) test system is an effective means to investigate the dynamic properties of rocks under impact loading. To further understand the failure characteristics and anisotropic dynamic properties of stratified rocks, SHPB tests have been performed on various materials, including coal, sandstone, and slate [20,21,22,23,24,25]. To determine the dynamic mechanical properties of shale, Sun et al. [26] conducted dynamic compression tests on seven groups of shale samples with varying bedding angles using an SHPB device. They investigated the effects of bedding angles on the failure modes, dynamic strength, and energy dissipation characteristics of shale at different strain rates, thereby revealing the damage mechanism of shale under dynamic loading. Fan et al. [23] investigated the dynamic compressive mechanical behavior and constitutive relationships of stratified shale using an SHPB apparatus and analyzed its dynamic properties, including compressive strength, failure strain, elastic modulus, and energy absorption. Guo et al. [27] performed a series of SHPB tests on shale with five bedding angles and observed that the dynamic tensile strength of shale increased with the loading rate. They identified the following three primary failure modes: tensile failure, shear failure, and tensile–shear failure.

Although the above studies indicate that the bedding structure greatly influences the mechanical properties of shale, systematic and comprehensive research on the dynamic compressive and tensile mechanical properties of shale remains scarce. Hence, the purpose of this paper is to identify the dynamic compressive and tensile mechanical properties and failure characteristics of shale using high-speed photography and digital image correlation technology (DIC) through the SHPB apparatus and to further clarify the anisotropy of shale’s dynamic mechanical properties and failure modes.

## 2. Experimental Methods

### 2.1. Shale Sample Selection and Preparation

**Figure 1:**
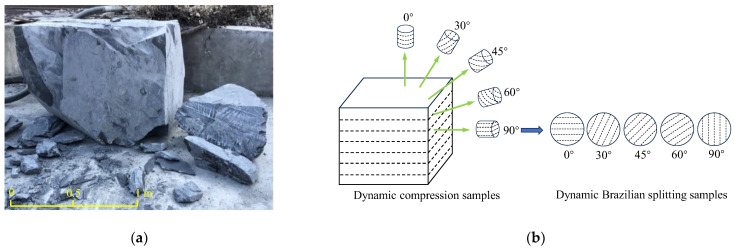
Schematic diagram of shale samples’ preparation: (**a**) large-sized shale blocks; (**b**) drilling and coring of shale samples.

**Figure 2:**
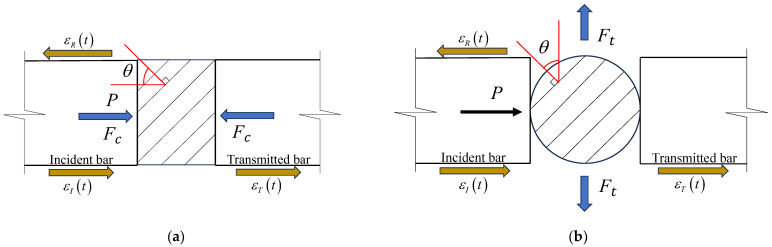
Schematic loading diagram of the shale samples and corresponding bedding angle: (**a**) dynamic compressive test; (**b**) dynamic Brazilian splitting test.

Large-sized shale blocks were collected from a transportation tunnel in a shale formation located in western Hubei Province, China, and used for subsequent core sampling along different directions, as shown in Figure 1a. In addition, Figure 1b illustrates a schematic diagram of the coring and sampling of the large-sized shale blocks, as well as the preparation of the shale samples for the dynamic compressive and Brazilian splitting tests at various bedding angles. The definition of the bedding angle of the prepared shale samples is shown in Figure 2. In the dynamic compressive tests, the angle between the dynamic compressive force, *F_c_*, and the normal line of the bedding planes of the shale samples is defined as the bedding angle, *θ*. For the dynamic Brazilian splitting tests, the angle between the actual dynamic tensile force, *F_t_*, and the normal line of the bedding planes of the shale samples was similarly defined as the bedding angle, *θ*. This unified definition facilitates a subsequent comparative analysis of the anisotropic mechanical behaviors under both compressive and tensile dynamic loading conditions. In order to systematically study the dynamic anisotropic mechanical properties of shale, five bedding angles *θ* of 0°, 30°, 45°, 60°, and 90° were set in both the dynamic compressive and Brazilian splitting tests. Based on the rock dynamics mechanics testing criteria of the International Society of Rock Mechanics (ISRM) [28], the prepared cylindrical shale samples for both the dynamic compressive and Brazilian splitting tests had a length-to-diameter (*L/D*) ratio of 2:1, a length (*L*) of 25 mm, and a diameter (*D*) of 50 mm. The two end surfaces of the prepared shale samples were polished to ensure that the flatness of the upper and lower surfaces was within ±0.02 mm and the end surfaces were perpendicular to the axis of the samples, with a permissible error of no more than ±0.25°.

### 2.2. Experimental Equipment

**Figure 3:**
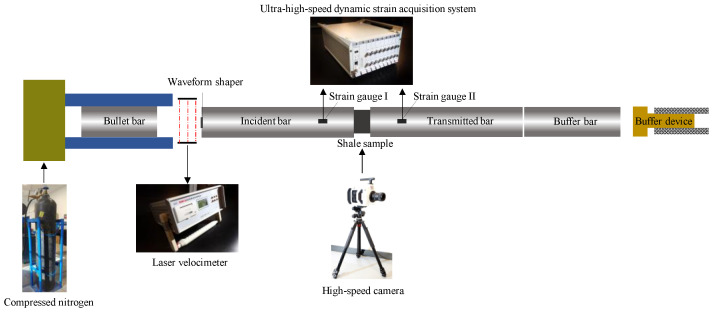
The SHPB test system.

The 50 mm diameter split Hopkinson pressure bar (SHPB) test system (shown in Figure 3) was used for this experiment. The system consists of a launching cavity, bullet bar, incident bar, transmitted bar, buffer end, laser velocimeter, and data logger. The basic bar parameters of the SHPB are as follows: The incident bar, transmitted bar, and bullet bar are made of high-strength alloy steel with a density of 7850 kg/m^3^ and modulus of elasticity of 210 GPa. The lengths of the incident rod and transmitted rod are 2500 mm, and the length of the bullet is 400 mm.

The impact velocity of the bullet is controlled by adjusting the air pressure of the high-purity nitrogen in the launch chamber, allowing the samples to achieve different strain rates and impact velocities. The contact surfaces of the rock samples with the incident and launching rods were coated with petroleum jelly (Sonneborn Superla^®^ Jelly, Sonneborn LLC, Petrolia, PA, USA; CAS 8009-03-8) to prevent a friction effect at the contact surfaces from affecting the distribution of the uniform stress inside the sample. In addition, a circular rubber gasket of 30 mm × 3 mm was pasted on the impact surface of the incident rod and the bullet to prolong the loading time of the samples and reduce the rising slope of the incident wave [29]. To record the failure process data of the shale samples under dynamic compressive and tensile loadings, the following integrated data acquisition system was adopted: first, a high-speed camera (Phantom, Wayne, NJ, USA; V1612, maximum frame rate of 620,000 fps, which was set at 100,000 fps for the experiment) captured the compressive and tensile failure processes of the shale samples and then a 4-channel, ultra-high-speed dynamic strain acquisition system (Qinhuangdao Longke Measurement and Control Technology Co., Ltd., Qinhuangdao, China; LK2109A, maximum sampling frequency of 50 MHz, which was set at 10 MHz for the experiment) synchronously collected dynamic strain data. In the dynamic Brazilian splitting test, a surface speckle was applied to the shale samples’ surfaces to enhance the image analysis features before dynamic loading. The digital image correlation (DIC) analyzed the high-speed photographic video (frame rate of 100,000 fps) of the dynamic tensile failure process of the shale samples after the surface speckle treatment to determine the surface strain concentration, clarifying the tensile failure characteristics.

### 2.3. Scheme and Principle of the Shale Dynamic Compressive Test

For the dynamic compressive test, the impact pressures were set to 0.1, 0.2, 0.3, and 0.4 MPa to achieve different shale sample strain rates with different bedding angles. To ensure statistical reliability, each experimental condition was replicated three times. During a test, the high-speed impact of the bullet bar on the incident bar induces an incident stress wave, which propagates along the axial direction of the incident bar and causes dynamic elastic deformation of the bar. The strain gauges attached to the surface of the incident bar measure the local strain changes, recording this wave signal as the incident dynamic strain, $\varepsilon_{I}\;\left(t\right)$, defined as the incident strain wave. When the incident stress wave reaches the interface between the incident bar and the shale sample, because of the difference in the wave impedance between the sample and the incident bar, part of the incident stress wave is reflected back into the incident bar for reverse propagation, forming a reflected stress wave. The dynamic elastic deformation of the incident bar caused by this reflection is recorded by the strain gauges on the bar as reflected dynamic strain, $\varepsilon_{R}\;\left(t\right)$, defined as the reflected strain wave. Additionally, another portion of the incident stress wave passes through the sample and enters the transmitted bar, forming a transmitted stress wave. The dynamic elastic deformation of the transmitted bar caused by this wave is recorded by the strain gauges on the bar as transmitted dynamic strain, $\varepsilon_{T}\;\left(t\right)$, defined as the transmitted strain wave, as shown in Figure 2a. By measuring the incident strain wave, $\varepsilon_{I}\left(t\right)$, the reflected strain wave, $\varepsilon_{R}\;\left(t\right),$ and the transmitted strain wave, $\varepsilon_{T}\;\left(t\right),$ at the front and back ends of the samples, the stress, $\sigma_{S}\;\left(t\right)$, strain rate, ${\dot{\varepsilon }}_{S}\;\left(t\right),$ and strain, $\varepsilon_{S}\;\left(t\right)$, of the samples are, respectively, determined according to the following three-wave method equations [30]:

(1) $$\sigma_{S}\left(t\right)=\frac{AE}{2A_{S}}\left[\varepsilon_{I}\left(t\right)+\varepsilon_{R}\left(t\right)+\varepsilon_{T}\left(t\right)\right]$$

(2) $${\dot{\varepsilon }}_{S}\left(t\right)=\frac{C_{0}}{l_{S}}\left[\varepsilon_{T}\left(t\right)-\varepsilon_{I}\left(t\right)+\varepsilon_{R}\left(t\right)\right]$$

(3) $$\varepsilon_{S}\left(t\right)=\int_{0}^{t}{\dot{\varepsilon }}_{S}\left(t\right)dt=\frac{C_{0}}{l_{S}}\int_{0}^{t}\left[\varepsilon_{T}\left(t\right)-\varepsilon_{I}\left(t\right)+\varepsilon_{R}\left(t\right)\right]dt$$

where *A*, *E,* and *C*_0_ are the cross-sectional area, elastic modulus, and longitudinal wave velocity of the elastic rods, respectively; *A*_s_ and *l*_s_ are the cross-sectional area and the length of the shale samples; and *t* is the strain wave propagation time. At the same time, based on the stress balance conditions at both ends of the samples in the SHPB test, the above Equations (1)–(3) can be simplified as two-wave method equations, as follows:

(4) $$\sigma_{S}\left(t\right)=\frac{AE}{A_{S}}\varepsilon_{T}\left(t\right)$$

(5) $${\dot{\varepsilon }}_{S}\left(t\right)=-2\frac{C_{0}}{l_{S}}\varepsilon_{R}\left(t\right)$$

(6) $$\varepsilon_{S}\left(t\right)=-2\frac{C_{0}}{l_{S}}\int_{0}^{t}\varepsilon_{R}\left(t\right)dt$$

Based on the three-wave or two-wave method equations [31,32,33,34], the dynamic stress–strain curves of the shale samples can be calculated from the recorded data of the incident, transmitted, and reflected strain waves on the incident and transmitted bars.

**Figure 4:**
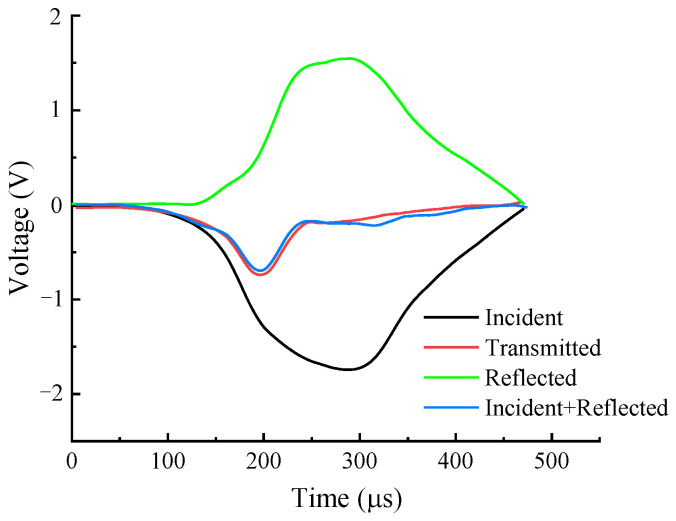
Verification of the dynamic stress balance of the shale sample under compressive loading.

**Figure 5:**
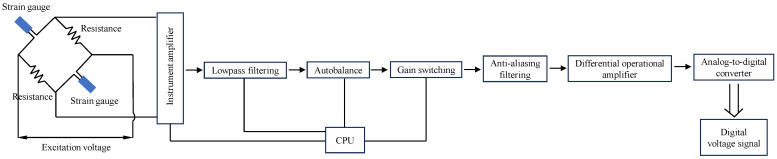
Schematic diagram of the measuring circuit.

To validate the dynamic stress equilibrium of the shale samples in the dynamic compressive test, the voltage–time curves from both ends of the shale samples were recorded and analyzed to confirm whether the dynamic stress equilibrium was maintained at the boundaries of the samples during dynamic compression. Figure 4 shows the incident wave, reflected wave, and transmitted wave in the form of voltage at both ends of the shale sample under typical test conditions. The recorded voltage waveforms were measured and recorded by the Wheatstone bridge, amplifier, and analog-to-digital converter (ADC) integrated into the ultra-high-speed dynamic strain acquisition system, as shown in Figure 5. The resistance changes in the strain gauges (ZEMIC, Xi’an, China; BE120-3AA) during the dynamic compression of the shale samples were converted into small voltage changes by a Wheatstone bridge and amplified by an amplifier. The amplified analog voltage signals were then digitized by an ADC at a sampling frequency of 10 MHz. In Figure 4, the superimposed voltage amplitudes of the incident and reflected waves at one end of the sample are consistent with those of the transmitted wave at the other end (error range of ±5%), indicating that the shale sample maintained dynamic stress equilibrium throughout the entire dynamic compressive process.

### 2.4. Scheme and Principle of the Dynamic Brazilian Splitting Test for the Shale

At present, the tensile strength of rock materials is mainly measured indirectly and the Brazilian splitting test is the main test method. The principle of the dynamic Brazilian splitting test is similar to that of the static Brazilian splitting test, which is generally carried out by a dynamic loading device such as the SHPB device. To investigate the influence of bedding angle and impact velocities on the dynamic tensile mechanical properties of shale, the impact pressures were set to 0.1, 0.2, and 0.3 MPa to achieve different impact velocities.

In Figure 2b, the diameter, length, and elastic modulus of the incident and the transmitted bar are *D*, *L*, and *E*, and the diameter of the shale samples is *D_S_*. In addition, the incident strain wave, $\varepsilon_{I}\;\left(t\right)$, the reflected strain wave, $\varepsilon_{R}\;\left(t\right),$ and the transmitted strain wave, $\varepsilon_{T}\;\left(t\right),$ were recorded during the dynamic tensile loading. Combined with the principle of the Brazilian splitting test under static loading and assuming that the maximum load borne by the shale sample during dynamic loading is *P_max_*(*t*), the dynamic tensile strength, *σ_td_*, of shale can be obtained using Equation (7) [35].

(7) $$\sigma_{td}=\frac{2P_{\max}(t)}{\pi DL}=\frac{ED^{2}}{2D_{S}L}{\left[\varepsilon_{i}(t)+\varepsilon_{r}(t)\right]}_{\max}=\frac{ED^{2}}{2D_{S}L}\varepsilon_{t}{\left(t\right)}_{\max}$$

If the diameter of the bars is equal to the diameter of the shale samples, the above formula can be simplified as Equation (8).

(8) $$\sigma_{td}=\frac{ED}{2L}{\left[\varepsilon_{i}(t)+\varepsilon_{r}(t)\right]}_{\max}=\frac{ED}{2L}\varepsilon_{t}{\left(t\right)}_{\max}$$

**Figure 6:**
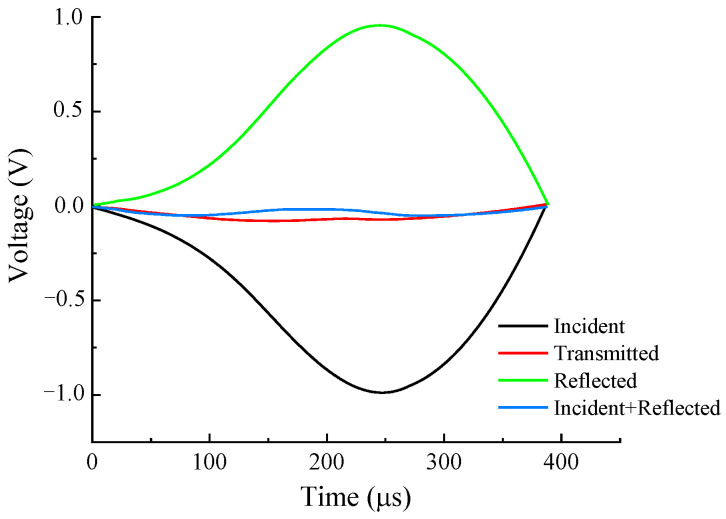
Verification of the dynamic stress balance of the shale sample under tensile loading.

To verify whether the dynamic stress equilibrium of the shale samples in the dynamic Brazilian splitting test was reached, the incident strain wave, reflected strain wave, and transmitted strain wave in the form of voltage at both ends of the shale sample under typical test conditions were collected as shown in Figure 6.

Figure 6 illustrates that the superimposed waveform of the incident strain wave and the reflected strain wave is basically consistent with that of the transmitted strain wave, indicating that the dynamic stress of the sample reached equilibrium during the dynamic splitting process.

## 3. Dynamic Compressive Mechanical Properties of Shale

### 3.1. Dynamic Stress–Strain Curves and Mechanical Parameters

**Figure 7:**
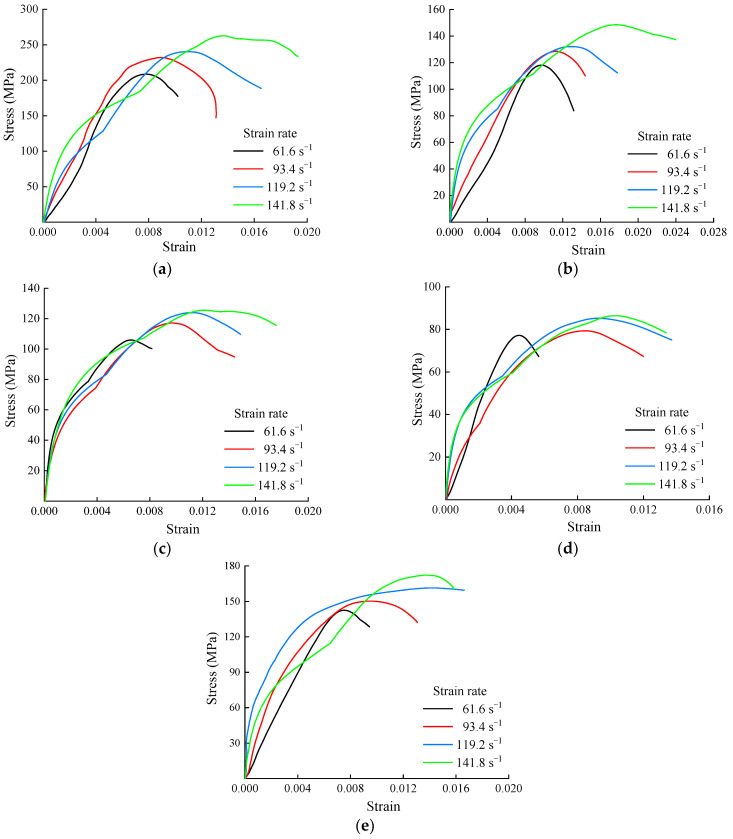
Dynamic stress–strain curves of the shale samples under different strain rates: (**a**) 0° bedding angle; (**b**) 30° bedding angle; (**c**) 45° bedding angle; (**d**) 60° bedding angle; (**e**) 90° bedding angle.

After the dynamic compressive test, the dynamic stress–strain curves of the shale samples with different bedding angles under various strain rates were calculated, as shown in Figure 7.

It is worth noting that the strain rates of the shale samples with different bedding angles under distinct impact pressures shown in Figure 7 were uniformly selected as 61.6, 93.4, 119.2, and 141.8 s^−1^, which were determined by the average strain rates of these samples under the same impact pressure, to facilitate the subsequent comparative analysis. As observed in Figure 7, at lower strain rates, the dynamic stress–strain curves of the shale samples exhibit a concave shape in the initial stage, indicating the presence of a compaction phase. In order to quantitatively analyze the influence of the strain rate and bedding angle on the dynamic mechanical properties of shale, the characteristic parameters of the dynamic stress–strain curves of the shale samples with different strain rates and bedding angles were compiled, as shown in Table 1 below.

**Figure 8:**
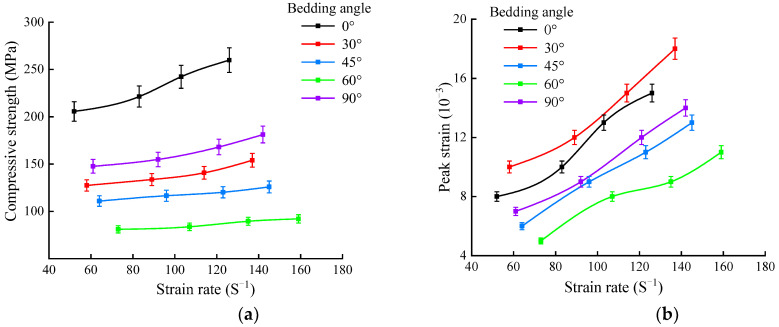
Variation in the dynamic compressive strength and peak strain with strain rate: (**a**) dynamic compressive strength; (**b**) peak strain.

Table 1 indicates that the impact velocity and strain rate of the samples increase with the impact pressure. As the strain rate increased, both the dynamic compressive strength and peak strain of the shale samples increased, as shown in Figure 8.

**Figure 9:**
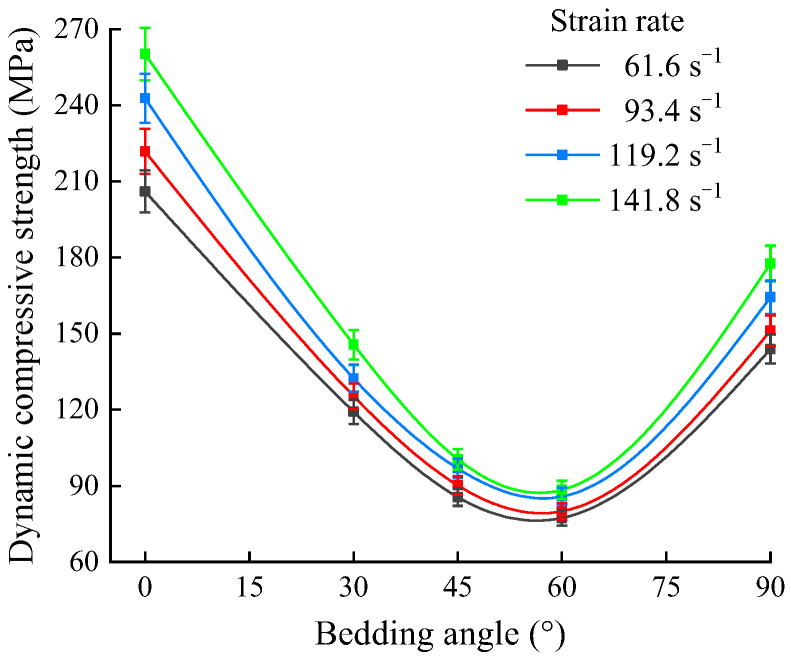
Variation in the dynamic compressive strength of shale at bedding angles.

The above data presented in Figure 8 and Table 1 suggest that as the strain rate increased, the dynamic compressive strength of the shale samples at a bedding angle of 0° exhibits the greatest increase, followed by samples at 30° and 90°, with the smallest increases observed at 45° and 60°. At bedding angles of 45° and 60°, the shale samples primarily underwent slip failure along the bedding planes, which affected the increase in their strength [36,37]. Conversely, at bedding angles of 0°, 30°, and 90°, the failure of the shale samples mainly occurred at the fracture planes of the rock matrix, thus resulting in a larger increase in dynamic compressive strength. Simultaneously, as the strain rate increased, the growth pattern of the peak strain of the shale samples was opposite to that of the compressive strength, specifically manifesting as a larger increase in the peak strain at bedding angles of 45° and 60°, and a smaller increase at bedding angles of 0°, 30°, and 90°. This was due to the significantly greater deformation during the slip failure of the samples compared to the tensile failure along the rock matrix. To analyze the influence of the bedding angle on the dynamic compressive strength, the dynamic compressive strength variation in the shale samples at bedding angles under different strain rates was compiled, as shown in Figure 9.

**Figure 10:**
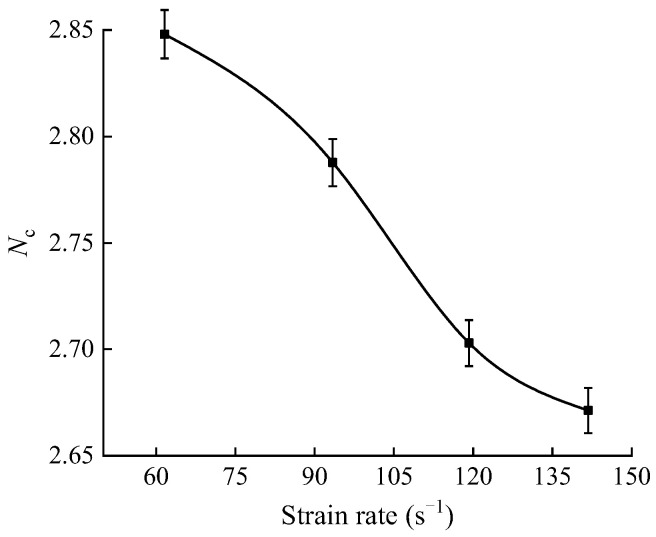
Dynamic compressive strength anisotropy coefficient of shale at different strain rates.

As seen in Figure 9, the dynamic compressive strength of shale follows an approximate U-shaped trend as the bedding angle increases. Specifically, the compressive strength of shale was relatively high at bedding angles of 0° and 90°, followed by angles of 30° and 45°, while it was minimal at a bedding angle of 60°. At bedding angles of 30°, 45°, and 60° the shale samples primarily experienced slip shear failure along the bedding planes, while at bedding angles of 0° and 90°, the shale samples mainly underwent shear and tensile splitting failure throughout the shale matrix. Since the mechanical strength of the bedding planes is significantly lower than that of the shale matrix, the overall strength of the shale decreases significantly when failure occurs along the bedding planes. To quantitatively study the degree of anisotropy in the dynamic compressive strength, the dynamic compressive strength anisotropy coefficient, denoted as *N*_c_, is defined by Equation (9).

(9) $$N_{c}=\frac{\sigma_{c(\beta =i,max)}}{\sigma_{c(\beta =j,min)}}$$

where $\sigma_{c(\beta =i,min)}\;$ and $\sigma_{c(\beta =j,min)}\;$are the maximum and minimum values of the average dynamic compressive strength of the shale samples with different bedding angles, and *i* and *j* are the bedding angles corresponding to the maximum and minimum values of the average compressive strength, respectively. The anisotropy coefficients of the dynamic compressive strength of shale under different strain rates are plotted in Figure 10.

As seen in Figure 10, the anisotropy coefficient of the dynamic compressive strength of shale decreases with the increase in the strain rate. Meanwhile, according to the classification of the anisotropy degree of rock mechanical parameters proposed by Singh et al. [38], the dynamic compressive strength of shale exhibits a moderate degree of anisotropy.

### 3.2. Dynamic Compressive Failure Mode

**Figure 11:**
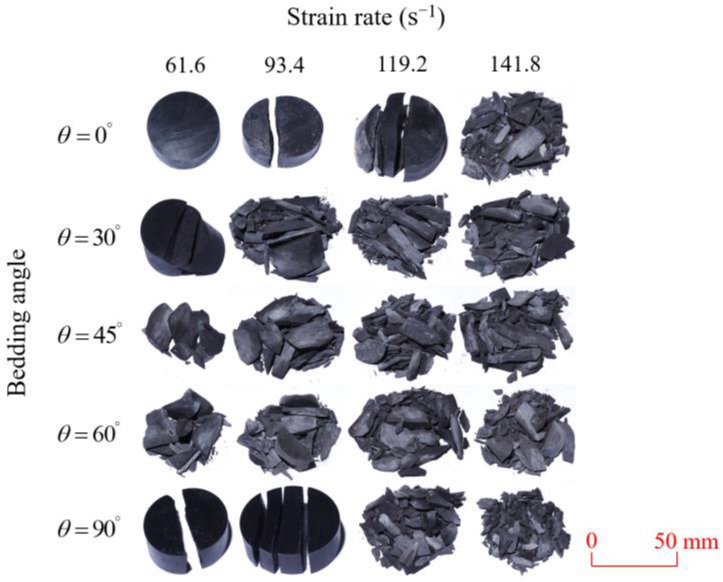
Dynamic compressive fragmented shale samples under varying strain rates.

Shale samples subjected to dynamic failure under various strain rates at different bedding angles are shown in Figure 11.

As observed in Figure 11, there was significant variation in the dynamic failure modes of the shale samples with different strain rates and bedding angles. The specimens with bedding angles of 45° and 60° exhibited a greater degree of failure compared to those with other bedding angles. When the strain rate was 61.6 s⁻^1^, the shale sample with a 0° bedding angle remained intact, while those with 30° and 90° bedding angles exhibited minor failure. In contrast, the samples with 45° and 60° bedding angles showed relatively more severe damage. As the strain rate increased, the degree of failure in these samples also escalated. When the strain rate reached 141 s⁻^1^, the shale samples with various bedding angles were all fully fragmented.

**Figure 12:**
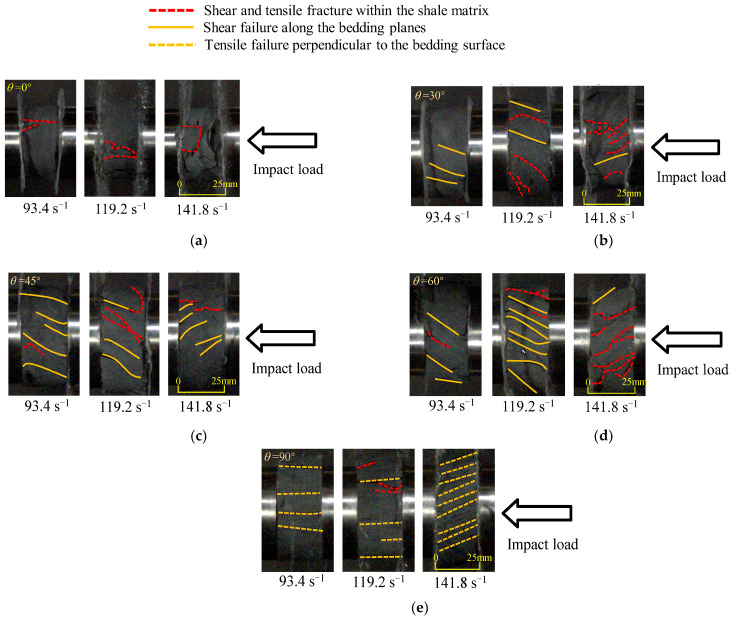
Characteristics of crack propagation during the impact compressive failure of shale samples under different strain rates. (**a**) 0° bedding angle; (**b**) 30° bedding angle; (**c**) 45° bedding angle; 306 (**d**) 60° bedding angle; (**e**) 90° bedding angle.

On the other hand, high-speed cameras (frame rates of 100,000 fps) were utilized to capture the crack propagation characteristics during the dynamic compression of the shale samples with five different bedding angles, under three strain rates (93.4, 119.2, and 141.8 s^−1^), as illustrated in Figure 12. Shale samples tested at a 61.6 s^−1^ strain rate had a lower degree of fragmentation, and the crack expansion was not obvious, so they are not analyzed here.

Figure 12 indicates that for shale samples with a bedding angle of 0°, the cracks within the samples under varying strain rates were primarily tensile cracks within the shale matrix. For samples with a bedding angle of 30°, the distribution of cracks changed with increasing strain rates. When the strain rate exceeded 119 s^−1^, the cracks in the samples transitioned from shear failure cracks along the bedding planes to primarily shear failure cracks along both the bedding planes and the shale matrix. For samples with bedding angles of 45° and 60°, the internal cracks were predominantly shear cracks along the bedding planes, penetrating throughout the entire samples. When the strain rate exceeded 119 s^−1^, several shear failure cracks appeared within the shale matrix. For samples with a bedding angle of 90°, the cracks in the samples under varying strain rates were primarily tensile failure cracks perpendicular to the bedding planes.

By combining Figure 11 and Figure 12, it can be observed that the failure modes of the shale samples with different bedding angles were as follows: For the samples with bedding angles of 0° and 90°, the primary failure modes at varying strain rates were characterized by tensile splitting within the shale matrix and along the bedding planes, respectively. For samples with bedding angles of 30°, 45°, and 60°, the predominant failure mode at low strain rates was shear failure along the bedding planes. However, as the strain rate increased, the failure mode transitioned to a composite shear failure along both the bedding planes and the shale matrix.

To quantitatively analyze the patterns of dynamic failure energy dissipation in the shale specimens under varying strain rates, based on one-dimensional stress wave theory and the principle of the SHPB test, the incident energy, *W_I_*, reflected energy, *W_R_*, and transmitted energy, *W_T_,* of the shale samples under dynamic compression are calculated as follows [39]:

(10) $$W_{I}\left(t\right)=ECA\int_{0}^{t_{1}}\varepsilon_{I}^{2}\left(t\right)dt$$

(11) $$W_{R}\left(t\right)=ECA\int_{0}^{t_{2}}\varepsilon_{R}^{2}\left(t\right)dt$$

(12) $$W_{T}\left(t\right)=ECA\int_{0}^{t_{3}}\varepsilon_{T}^{2}\left(t\right)dt$$

where *A*, *E,* and *C* represent the cross-sectional area, elasticity modulus, and longitudinal wave speed of elastic bars, respectively, and *t*_1_, *t*_2_, and *t*_3_ represent the duration of the initial wave of the incident, reflected, and transmitted strain waves, respectively, in each shale sample during the dynamic compressive test.

The energy absorbed, *W_S_*, by the shale sample includes the energy consumed for sample fracturing, *W_FD_*, the kinetic energy, *W_K_*, of the post-fracturing fragments, and other forms of energy dissipation, *W_O_*, such as acoustic, thermal, and radiation energy. Among these components, *W_FD_* accounts for approximately 95% of *W_S_*, while *W_K_* and *W_O_* are negligible and can be omitted [40,41]. Thus, the absorbed energy, *W_S_*, of the shale sample can be considered approximately equal to the fracturing energy, *W_FD_*, which implies that the absorbed energy of the sample is primarily used for fracturing and can be calculated as Equation (13).

(13) $$W_{S}\left(t\right)=W_{I}\left(t\right)-W_{R}\left(t\right)-W_{T}\left(t\right)$$

To quantitatively analyze the energy dissipation patterns of the shale samples under varying strain rates, the energy absorption rate, *η,* is defined as the ratio of absorbed energy, *W_S_*, to incident energy, *W_I_*, as shown in Equation (14).

(14) $$\eta =\frac{W_{S}\left(t\right)}{W_{I}\left(t\right)}$$

**Figure 13:**
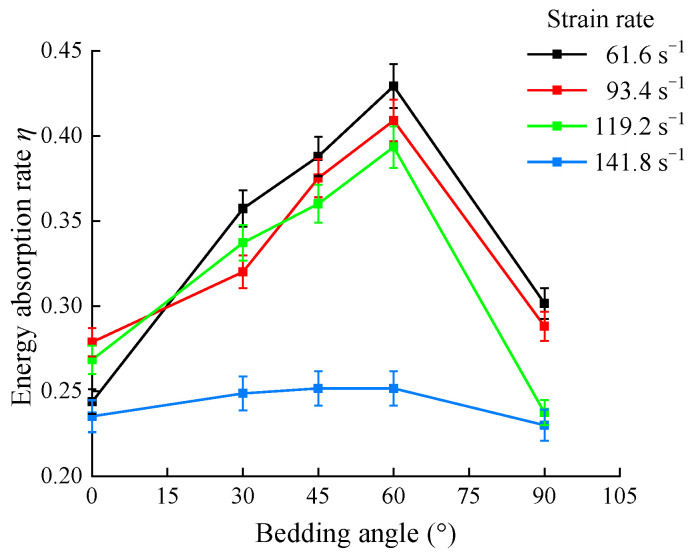
Energy dissipation patterns of shale under varying strain rates.

The energy absorption rates, *η*, of the shale samples were calculated and plotted as shown in Figure 13.

As observed in Figure 13, the energy absorption rate, *η,* of the shale samples under different strain rates initially increased and then decreased with the increase in the bedding angle, with the maximum energy absorption rate occurring at a bedding angle of 60°. When the strain rates were 61.6, 93.4, and 119.2 s^−1^, the energy absorption rates of the samples with bedding angles of 30°, 45°, and 60° were relatively high. Correspondingly, the dynamic fragmentation degree of the shale samples was also high, indicating that the greater the energy absorption of the shale samples, the greater their fragmentation degree. The shale samples with 0° and 90° bedding angles demonstrated comparatively lower energy absorption rates relative to those with 30°, 45°, and 60° bedding angles, while also exhibiting correspondingly reduced failure levels. When the strain rate reached 141.8 s⁻^1^, the shale samples were fully fragmented. However, compared to the strain rate of 119.2 s⁻^1^, the degree of fragmentation of the samples did not change significantly, while their absorbed energy was primarily utilized for fragmentation, resulting in a limited increase in their absorbed energy. However, the incident energy was exclusively influenced by the impact velocity, with an increase in the impact velocity resulting in a higher strain rate and, consequently, greater incident energy. When the strain rate increased from 119.2 to 141.8 s⁻^1^, the incident energy of the samples continued to increase, thereby inducing a decrease in their energy absorption rate. Concurrently, at a strain rate of 141.8 s⁻^1^, the energy absorption rates of the shale samples with different bedding angles were relatively close, ranging from 0.2 to 0.25. At this point, the correlation between the degree of the sample fragmentation and the bedding angle decreased, indicating that the energy absorption rates of the shale samples with different bedding angles began to converge.

## 4. Dynamic Tensile Mechanical Properties of Shale

### 4.1. Dynamic Tensile Strength Analysis

Based on the incident strain wave, reflected strain wave, and transmitted strain wave collected in the dynamic Brazilian splitting test, combined with Equations (7) and (8), the average dynamic tensile strengths of the shale with different bedding angles under varying impact velocities were calculated, as presented in Table 2.

**Figure 14:**
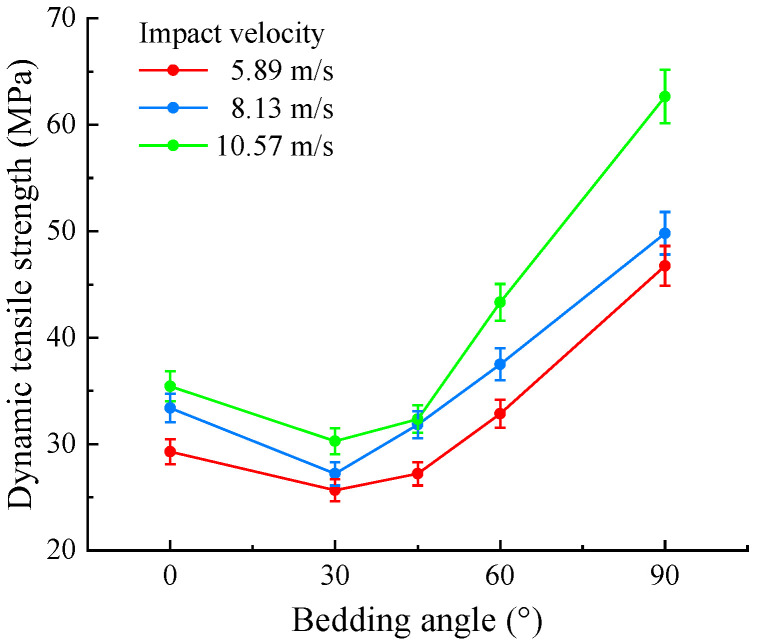
Variation in the dynamic tensile strength with the bedding angle.

As indicated in Table 2, under the same impact pressure, the impact velocities of the bullets were relatively close. When the impact pressures were 0.1, 0.2, and 0.3 MPa, the average impact velocities corresponded to 5.89, 8.13, and 10.57 m/s, respectively. We also observed that under the same impact pressure, the dynamic tensile strength was greatest when the bedding angle was 90°, followed by 0°, 45°, and 60°, with the smallest value occurring at a bedding angle of 30°. To visually analyze the variation pattern of shale’s dynamic tensile strength with the bedding angle, the dynamic tensile strengths under different average impact velocities were collated, as shown in Figure 14.

As seen in Figure 14, under varying impact velocities, the dynamic tensile strength exhibited a trend of initially decreasing followed by an increase as the bedding angle increased, which is consistent with the variation in the dynamic compressive strength. The dynamic tensile strength was minimal when the bedding angle was 30° and reached its maximum when the bedding angle was 90°. As the bedding angle increased from 0° to 30°, the dynamic tensile strength of the shale gradually decreased. When the bedding angle increased to 45°, the dynamic tensile strength slightly increased. However, as the bedding angle increased from 45° to 90°, the dynamic tensile strength sharply increased. Additionally, based on Figure 14, we observed that as the impact velocity increased, the dynamic tensile strength of the shale at different bedding angles correspondingly increased.

**Figure 15:**
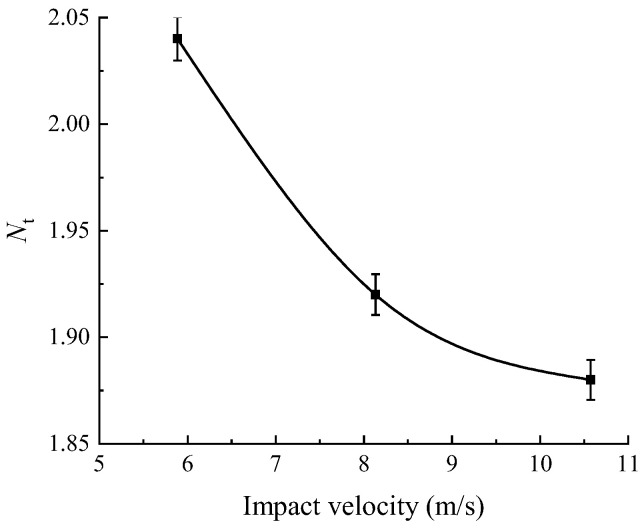
Anisotropy coefficient of the dynamic tensile strength of shale.

To quantitatively analyze the degree of anisotropy in the dynamic tensile strength of the shale, the anisotropy coefficient, *N*_t_, of the dynamic tensile strength of shale is defined as the ratio of the maximum to the minimum dynamic tensile strength of shale at different bedding angles under a certain average impact velocity, as expressed by Equation (15).

(15) $$N_{t}=\frac{\sigma_{t(\beta =i,max)}}{\sigma_{t(\beta =j,min)}}$$

where $\sigma_{t(\beta =i,min)}$ and $\sigma_{t(\beta =j,min)}\;$are the maximum and minimum values of the average value of the dynamic tensile strength of the shale samples with different bedding angles, and *i* and *j* are the bedding angles corresponding to the average tensile strength of the maximum and minimum values, respectively. Based on this, the anisotropy coefficients of the dynamic tensile strength of shale under different average impact velocities were plotted, as shown in Figure 15.

As indicated in Figure 15, the anisotropy coefficient of the dynamic tensile strength of shale varied between 1.88 and 2.04 under different average impact velocities. According to [35], the dynamic tensile strength of the shale exhibits a moderate to low degree of anisotropy. Furthermore, as the impact velocity increases, the anisotropy coefficient of the dynamic tensile strength decreases, indicating a corresponding reduction in the degree of anisotropy.

### 4.2. Dynamic Tensile Failure Mode

**Figure 16:**
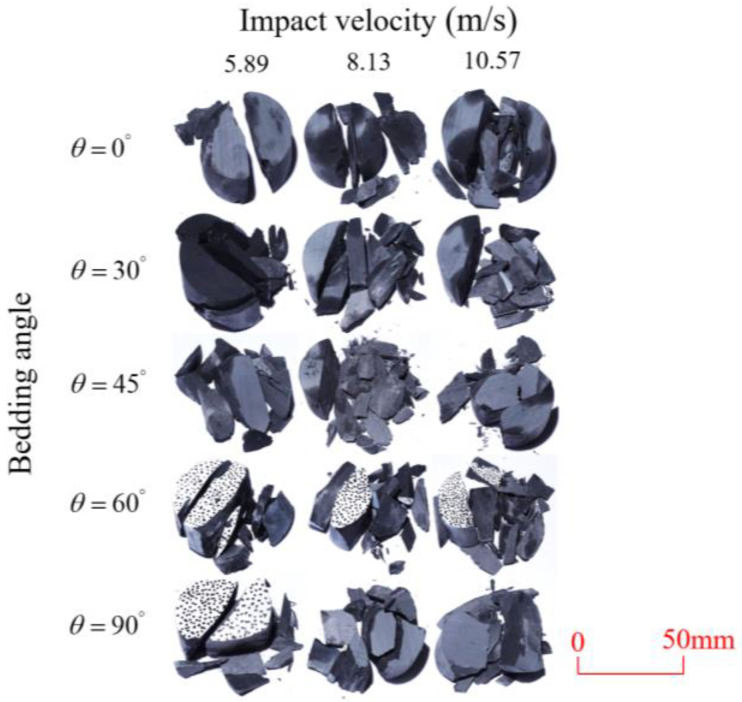
Dynamic tensile fragmented shale samples under varying impact velocities.

After completing the dynamic Brazilian splitting test, the shale samples subjected to dynamic tensile failure at various impact velocities and bedding angles were collected, as illustrated in Figure 16.

As seen in Figure 16, the characteristics of the shale samples after dynamic tensile failure under varying impact velocities are distinct. As the impact velocity increased, the degree of tensile failure in the shale samples also escalated. When the impact velocity reached 10.57 m/s, the samples were fully fragmented. Furthermore, the bedding angle also influenced the dynamic tensile failure characteristics of the shale samples. At the same impact velocity, samples with bedding angles of 30° and 45° demonstrated significantly greater degrees of failure compared to those with other bedding angles.

Compared to traditional electrical measurement methods, which can only detect the local strain information of an object, optical full-field measurement techniques can compensate for these limitations, acquiring comprehensive deformation data on the surface of the specimen, thus facilitating a more intuitive mechanical analysis. Digital image correlation (DIC), the primary non-contact optical measurement system technology, is utilized for the measurement and analysis of full-field displacement and strain on the surface of an object, obtaining the strain field distribution characteristics of the surface tested. Based on this, DIC technology was applied to the dynamic Brazilian splitting test, allowing for the direct observation of the distribution of dynamic strain on the surface of the shale specimen, thereby studying the characteristics of crack propagation during the dynamic tensile failure. The DIC analysis software employed in the experiment was only capable of two-dimensional planar dynamic strain field analysis. However, during the dynamic compression processes, surface curvature existed in the crack propagation area of the shale samples. This geometric characteristic limited the effectiveness of the DIC software (XTOP, Shenzhen, China; XTDIC, CONST-SD) in analyzing the dynamic strain fields on sample surfaces during the dynamic compression tests.

Based on high-speed photography videos of the dynamic splitting failure process of the shale samples after surface speckle processing, the evolution of the dynamic strain field on the sample surface was obtained through DIC analysis software. (The parameters of the high-speed camera system used for DIC analysis software were as follows: a resolution of 320 × 280 pixels and a sampling frequency of 100,000 fps.) The original images and DIC-processed images of the dynamic tensile failure crack breakthrough moments of these shale samples under different bedding angles and impact velocities were collated and are listed in Table 3. Support information can can be found in Supplementary Materials.

Based on the experimental results in Figure 16 and Table 3, it can be inferred that the bedding angle and impact velocity significantly influence the dynamic tensile failure mode of shale. When the bedding angle was 0°, multiple dynamic tensile cracks parallel to the loading direction emerged in the middle of the shale samples, forming a distinct tensile fracture zone. In conjunction with the DIC images, the dynamic strain concentration area transitioned from the incident end to the transmitted end as the impact velocity increased, ultimately distributing uniformly along the line connecting the incident and transmitted ends. Under this bedding angle, the shale exhibited tensile failure characteristics along the bedding plane. When the bedding angle was 30°, the shale specimens uniformly exhibit a combined tensile and shear failure along the bedding plane. At lower impact velocities, only a single dynamic tensile–shear crack appeared along the bedding plane in the upper part of the specimen. However, as the impact velocity increased, dynamic tensile–shear cracks appeared in both the upper and lower parts of the specimen. At higher impact velocities, a tensile–shear fracture zone emerged in the middle of the specimen along the bedding plane. When the bedding angle was 45°, the dynamic failure mode was similar to that at a bedding angle of 30°, with the difference being that as the impact velocity increased, no tensile–shear fracture zone appeared in the middle of the specimen, and tensile–shear cracks also emerged along the bedding plane and within the shale matrix at the lower end. At a bedding angle of 60°, the shale samples underwent a tensile–shear failure along the bedding planes at lower impact velocities. As the impact velocity increased, tensile–shear failure occurred both along the bedding plane and within the shale matrix. However, as the impact velocity continued to increase, tensile failure within the shale matrix became dominant, and the fracture surface no longer extended along the bedding plane. At a bedding angle of 90°, the impact velocity also significantly affected the dynamic tensile failure characteristics of shale specimens. At lower impact velocities, tensile–shear failure occurred along the bedding plane and within the shale matrix. As the impact velocity increased, the specimens mainly underwent tensile failure along the loading direction within the shale matrix.

## 5. Comparison of the Dynamic Compressive and Tensile Mechanical Properties and Failure Modes of Shale

**Figure 17:**
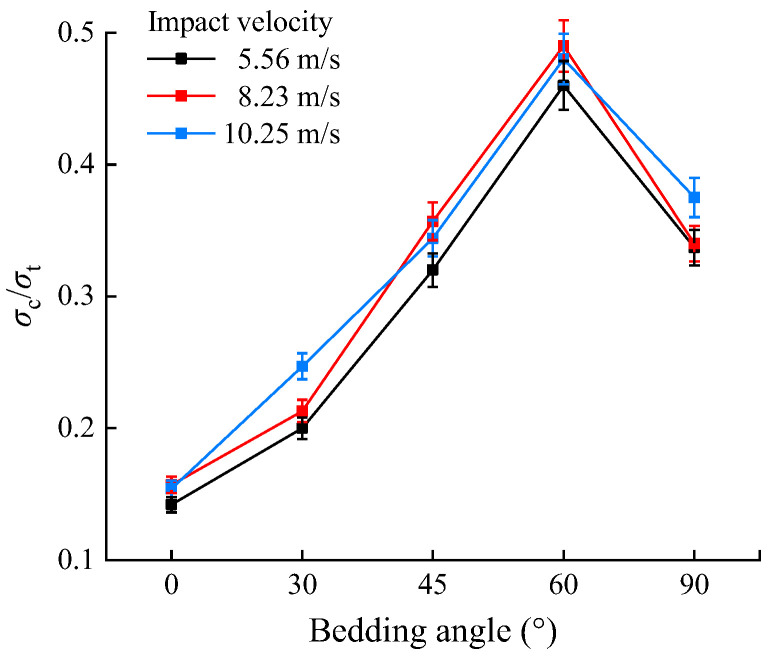
Ratio of the dynamic tensile strength to the dynamic compressive strength of shale samples at different impact velocities.

In the dynamic compression tests, the average impact velocities of the shale samples were 5.22, 8.33, 10.3, and 12.25 m/s. Conversely, in the dynamic Brazilian splitting tests, the corresponding average impact velocities of the samples were 5.89, 8.13, and 10.57 m/s. Based on the similar impact velocities in the two tests, the average velocities (5.56, 8.23, and 10.45 m/s) were selected as the reference for a comparative analysis of the test results. Furthermore, the variation pattern of the ratio of dynamic tensile strength to dynamic compressive strength of shale at different bedding angles under different average velocities was plotted, as illustrated in Figure 17.

As shown in Figure 17, the dynamic tensile strength was significantly lower than the dynamic compressive strength, with the ratio generally ranging from 0.1 to 0.5. Furthermore, when the bedding angles were 0°, 30°, 45°, 60°, and 90°, the mean ratios of dynamic tensile strength to dynamic compressive strength were approximately 0.15, 0.22, 0.34, 0.48, and 0.35, respectively. This indicates that as the bedding angle increased from 0° to 90°, the ratio of dynamic tensile strength to dynamic compressive strength under different impact velocities first increased and then decreased, reaching its maximum at a bedding angle of 60°. At this angle, the dynamic tensile strength reached an intermediate value, while the dynamic compressive strength was the smallest, resulting in a relatively larger ratio compared to other bedding angle conditions. The ratios of dynamic tensile strength to dynamic compressive strength for different bedding angles under varying impact velocities were relatively close, suggesting that at the same bedding angle, this ratio was less affected by the impact velocity.

**Figure 18:**
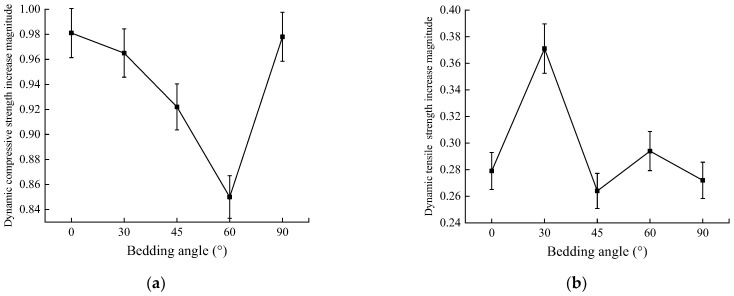
Variation in the increases in the magnitude of the dynamic strength at different bedding angles: (**a**) dynamic compressive strength; (**b**) dynamic tensile strength.

As the impact velocity increased from 5.56 to 10.45 m/s, the increases in the magnitudes of the dynamic compressive strength and tensile strength at different bedding angles also exhibited significant differences, and the variation patterns are illustrated in Figure 18.

As shown in Figure 18, when the impact velocity increased from 5.56 to 10.45 m/s, the increase in the range of the dynamic tensile strength at different bedding angles was 0.27 to 0.37, while the increase in the range of the dynamic compressive strength was 0.85 to 0.98. The increase in dynamic compressive strength was 2.3 to 3.6 times higher than that in the dynamic tensile strength, indicating that the sensitivity of the dynamic compressive strength to impact velocity was significantly higher than that of the dynamic tensile strength. Additionally, as seen in Figure 10 and Figure 15, under impact velocities of 5.56, 8.23, and 10.45 m/s, the anisotropy coefficients of the dynamic compressive strength were 2.79, 2.70, and 2.67, respectively, while those of dynamic tensile strength were 2.04, 1.92, and 1.88, respectively. Under the same impact velocities, the anisotropy coefficients of tensile strength were consistently lower than those of compressive strength, suggesting that the sensitivity of the dynamic compressive strength to the bedding angles was also stronger. Furthermore, as the impact velocity increased, the anisotropy coefficients of both the dynamic compressive strength and dynamic tensile strength showed a decreasing trend (with a 4.3% reduction for compressive strength and a 7.8% reduction for tensile strength). The decrease in the anisotropy coefficient of the dynamic tensile strength was more significant, indicating that as the impact velocity increased, the difference between the dynamic compressive strength and the dynamic tensile strength in sensitivity to bedding angles became more pronounced.

Regarding the dynamic compressive and dynamic tensile failure modes of shale, in dynamic compression tests, at lower impact velocities, the failure mode of the samples exhibited a phased change as the bedding angle increased from 0° to 90°, as follows: It gradually transitioned from tensile failure within the shale matrix (at a bedding angle of 0°) to shear failure along the bedding planes (at bedding angles of 30°, 45°, and 60°), ultimately evolving into tensile failure along the bedding planes (at a bedding angle of 90°). Furthermore, as the impact velocity increased, the failure modes of the shale samples at bedding angles of 0° and 90° remained unchanged, while those at bedding angles of 30°, 45°, and 60° gradually evolved into a composite shear failure along the bedding planes and within the shale matrix. Accompanying the transition in failure mode, the dynamic compressive strength of the shale samples exhibited a pattern of decreasing first and then increasing, with the dynamic compressive strength minimal at a bedding angle of 60° and maximal at a bedding angle of 0°.

In the dynamic Brazilian splitting test, when the impact velocity was relatively low, the failure mode of the samples also exhibited a staged evolution as the bedding angle increased from 0° to 90°, transitioning from tensile failure along the bedding planes (at a bedding angle of 0°) to a combination of tensile and shear failure along the bedding planes (at bedding angles of 30° and 45°), and finally evolving into a combination of tensile and shear failure along both the bedding planes and the shale matrix (at bedding angles of 60° and 90°). Additionally, as the impact velocity increased, the failure modes of the shale samples with bedding angles of 0°, 30°, and 45° remained unchanged, while the failure modes of samples with bedding angles of 60° and 90° gradually transformed into tensile failure within the shale matrix. Accompanying these changes in the failure modes, the dynamic tensile strength of the shale also showed a trend of first decreasing and then increasing. The dynamic tensile strength was minimized at a bedding angle of 30° and maximized at a bedding angle of 90°.

Comprehensive analysis of the results of the dynamic compression and dynamic Brazilian splitting tests revealed a significant correlation between the dynamic strength characteristics of the shale samples and their failure modes. When the failure mode of the shale samples was dominated by shear failure along the bedding planes (e.g., at a bedding angle of 60° in dynamic compression tests and 30° in dynamic Brazilian splitting tests), the dynamic strength of the shale was minimized. Conversely, when the failure mode was dominated by failure within the shale matrix (e.g., at a bedding angle of 0° in dynamic compression tests and 90° in dynamic Brazilian splitting tests), the dynamic strength of the shale was maximized.

## 6. Conclusions

(1) The dynamic compressive strength and tensile strength of the shale increases with the increases in the strain rate and impact velocity, and the dynamic compressive strength demonstrated a significantly higher sensitivity to both the strain rate and impact velocity compared to the dynamic tensile strength. With the increase in bedding angle, the dynamic strength of the shale exhibited a pattern of an initial decrease followed by increase because the minimum value of dynamic compressive strength is achieved at a bedding angle of 60°, while the minimum value of the dynamic tensile strength was achieved at a bedding angle of 30°. This indicates that the dynamic strength of the shale exhibited significant anisotropy. Furthermore, as the strain rate and impact velocity increased, the degree of anisotropy in the dynamic mechanical strength of the shale decreased.
(2) The dynamic compressive failure mode of the shale also exhibited anisotropic characteristics and was closely related to the strain rate. For the shale samples with bedding angles of 0° and 90°, the dynamic compressive failure modes under different strain rates primarily consist of splitting tensile failure within the shale matrix and along the bedding planes, respectively. For samples with bedding angles of 30°, 45°, and 60°, the failure mode was primarily shear failure along the bedding planes at low strain rates and evolved into a composite shear failure along both the bedding planes and the shale matrix as the strain rate increased.
(3) The dynamic tensile failure mode of the shale was influenced by the bedding angle and impact velocity. For the shale samples with a bedding angle of 0°, the dynamic tensile failure mode is characterized by tensile failure along the bedding planes. For samples with bedding angles of 30° and 45°, the primary failure mode involved a combination of tensile and shear failure along the bedding planes. For samples with bedding angles of 60° and 90°, the tensile and shear failure occurred along the bedding planes and within the shale matrix at lower impact velocities. However, at higher impact velocities, tensile failure along the loading direction within the shale matrix predominates.
(4) The dynamic strength characteristics of the shale samples were significantly correlated with their failure modes. When the failure mode was dominated by shear failure along the bedding planes (e.g., at a bedding angle of 60° in the dynamic compression tests and 30° in the dynamic Brazilian splitting tests), the dynamic strength of the shale was the lowest. Conversely, when the failure mode was dominated by failure within the shale matrix (e.g., at a bedding angle of 0° in the dynamic compression tests and 90° in the dynamic Brazilian splitting tests), the dynamic strength of the shale was the greatest.

## Figures and Tables

**Table 1:** Characteristic parameters of the dynamic stress–strain curves of shale samples with different strain rates and bedding angles.

Bedding Angle (°)	Impact Pressure (MPa)	Impact Velocity (m/s)	Compressive Strength (MPa)	Peak Strain (10^−3^)	Strain Rate (s^−1^)
0	0.1	5.16	206.08	8	52
	0.2	8.16	221.79	10	83
	0.3	10.13	242.75	13	103
	0.4	12.36	260.21	15	126
30	0.1	5.42	119.20	10	58
	0.2	8.39	125.31	12	89
	0.3	10.23	132.44	15	114
	0.4	12.39	145.69	18	137
45	0.1	5.23	85.53	6	64
	0.2	8.42	90.15	9	96
	0.3	10.34	96.90	11	123
	0.4	11.98	100.52	13	145
60	0.1	5.13	72.36	5	73
	0.2	8.35	79.56	8	107
	0.3	10.24	89.81	9	135
	0.4	12.44	97.41	11	159
90	0.1	5.14	143.96	7	61
	0.2	8.35	151.16	9	92
	0.3	10.67	164.36	12	121
	0.4	12.07	177.56	14	142

**Table 2:** Results of the dynamic Brazilian splitting test.

Bedding Angle (°)	Impact Pressure (MPa)	Impact Velocity (m/s)	Dynamic Tensile Strength (MPa)
0°	0.1	5.81	29.27
	0.2	8.01	34.88
	0.3	10.61	37.43
30°	0.1	6.08	23.81
	0.2	8.29	26.70
	0.3	10.24	32.65
45°	0.1	5.85	27.33
	0.2	8.37	32.19
	0.3	10.31	34.54
60°	0.1	5.92	33.32
	0.2	7.86	38.99
	0.3	10.70	43.32
90°	0.1	5.77	48.46
	0.2	8.14	51.35
	0.3	11.01	61.62

**Table 3:** Dynamic tensile failure original and DIC-processed images of the shale samples. Notes: The color scale on the right side of the DIC analysis diagram in Table 3 represents the distribution characteristics of the dynamic strain field on the sample surface. Purple and red regions correspond to the minimum and maximum strain levels, respectively, with numeric labels on the color scale annotated in percentage units to indicate the strain values associated with each color zone.

Bedding Angle (°)		Impact Velocity (m/s)		
		5.89	8.13	10.57
0	Original image	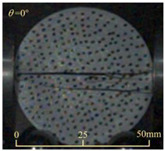	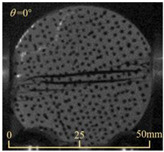	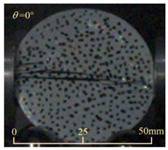
	DIC image	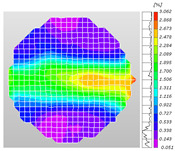	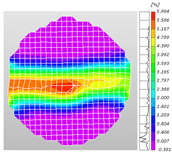	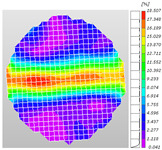
30	Original image	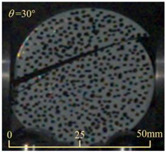	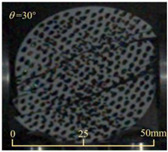	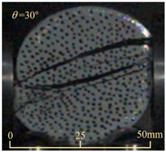
	DIC image	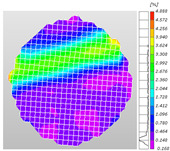	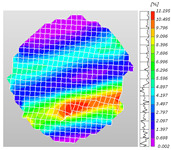	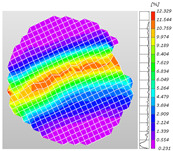
45	Original image	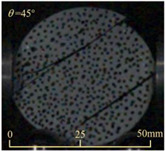	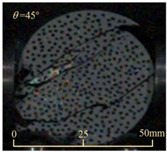	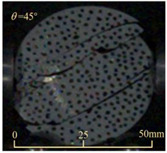
	DIC image	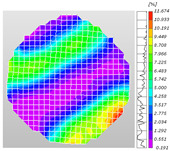	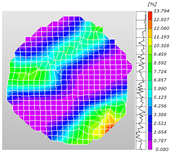	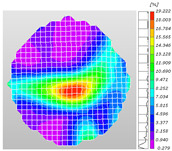
60	Original image	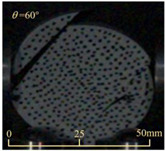	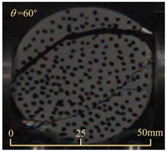	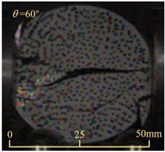
	DIC image	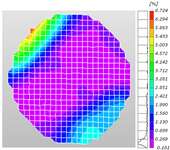	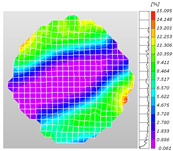	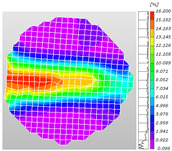
90	Original image	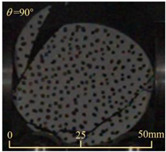	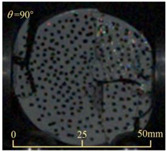	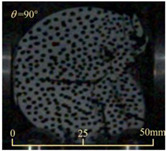
	DIC image	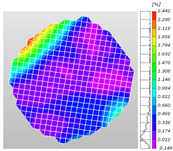	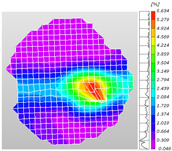	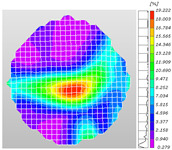

## Data Availability

The data used to support the findings of this study are available from the corresponding author upon request.

## Supplementary Materials

The following supporting information can be downloaded at: https://www.mdpi.com/article/10.3390/s25092905/s1, Figure S1: DIC images, Figure S2: Simple images, Video S1: DIC videos.
